# Characteristics and Prediction of RNA Structure

**DOI:** 10.1155/2014/690340

**Published:** 2014-07-06

**Authors:** Hengwu Li, Daming Zhu, Caiming Zhang, Huijian Han, Keith A. Crandall

**Affiliations:** ^1^School of Computer Science and Technology, Shandong Provincial Key Laboratory of Digital Media Technology, Shandong University of Finance and Economics, Jinan 250014, China; ^2^Computational Biology Institute, George Washington University, Ashburn, VA 20147, USA; ^3^School of Computer Science and Technology, Shandong Provincial Key Laboratory of Software Engineering, Shandong University, Jinan 250101, China

## Abstract

RNA secondary structures with pseudoknots are often predicted by minimizing free energy, which is NP-hard. Most RNAs fold during transcription from DNA into RNA through a hierarchical pathway wherein secondary structures form prior to tertiary structures. Real RNA secondary structures often have local instead of global optimization because of kinetic reasons. The performance of RNA structure prediction may be improved by considering dynamic and hierarchical folding mechanisms. This study is a novel report on RNA folding that accords with the golden mean characteristic based on the statistical analysis of the real RNA secondary structures of all 480 sequences from RNA STRAND, which are validated by NMR or X-ray. The length ratios of domains in these sequences are approximately 0.382*L*, 0.5*L*, 0.618*L*, and *L*, where *L* is the sequence length. These points are just the important golden sections of sequence. With this characteristic, an algorithm is designed to predict RNA hierarchical structures and simulate RNA folding by dynamically folding RNA structures according to the above golden section points. The sensitivity and number of predicted pseudoknots of our algorithm are better than those of the Mfold, HotKnots, McQfold, ProbKnot, and Lhw-Zhu algorithms. Experimental results reflect the folding rules of RNA from a new angle that is close to natural folding.

## 1. Introduction

RNAs are versatile molecules. Messenger RNAs carry genetic information and act as the intermediary agent between DNAs and proteins; ribosomal RNAs, transfer RNAs, and other noncoding RNAs also have important structural, regulatory, and catalytic functions in cells. To completely understand the various functions of RNAs, we need to first understand their structures. The primary structure of RNA is the sequence of nucleotides (i.e., four bases A, C, G, and U) in the single-stranded polymer of RNA. However, these sequences are not simply long strands of nucleotides. In RNA, complementary bases of guanine and cytosine pair can form three hydrogen bonds, those of adenine and uracil pair can form two hydrogen bonds, and those of guanine and uracil pair can form two hydrogen bonds. RNA folds into a 3D structure through hydrogen bonding and base stacking, which are nonconsecutive in the sequence. Noncanonical pairing and base-to-backbone hydrogen bonding also stabilize folding. The 3D arrangement of atoms in a folded RNA molecule is the tertiary structure; the collection of base pairs in the tertiary structure is the secondary structure. Experimental determination of RNA tertiary structures is too expensive and time consuming to meet practical needs; thus, predicting RNA structure by computer becomes a basic method and issue in computational biology [1].

The secondary structures of RNA include the scaffold of the tertiary structures. Predicting RNA secondary structures is the first step in predicting RNA tertiary structures from RNA sequences. Computational approaches for predicting RNA secondary structures can be classified into three families: thermodynamic, comparative, and hybrid. Thermodynamic approaches use dynamic programming to compute the optimal secondary structure for a single RNA sequence with globally minimal free energy [2], based on a set of experimentally determined energy parameters [3]. Such methods have been successful for relatively short RNAs. Manually comparative approaches are more reliable than thermodynamic approaches when many homologous sequences are available. Manually comparative approaches have been used to establish the structures of known RNA families. These approaches compute a consensus structure on a set of aligned RNA sequences by searching for covariance evidence between each of the base pairs. Quantitative measures of covariance have been implemented in *χ*2 statistics and mutual information. Akmaev et al. [4] also extended these approaches to explicitly consider sequence phylogeny and showed positive results. Hybrid approaches, which have recently emerged, combine the advantages of thermodynamic and comparative approaches [5]. Hybrid approaches consider both thermodynamic stability and sequence covariance and produce positive results on as few as three homologous sequences. Other methods cannot be classified into any of these three families. A few of these methods attempt to simultaneously align and fold homologous sequences [6]. Eddy and Durbin [7] introduced stochastic context-free grammars to align homologous sequences iteratively and found a consensus structure for them.

A pseudoknot motif is a prevalent RNA structure. Pseudoknots serve various functions in biology [1]. Plausible pseudoknotted structures have been proposed and confirmed for the 3′-end of several plant viral RNAs, where pseudoknots are apparently used to mimic tRNA structures. Pseudoknots have been recently confirmed in some RNAs of humans and other species [6].

Current studies on RNA secondary structure prediction have not considered pseudoknots. Optimizing secondary structures, including arbitrary pseudoknots, is NP-hard [8].

Most RNA folding methods that can fold pseudoknots adopt heuristic search procedures and sacrifice optimality. Examples of these methods include quasi-Monte Carlo searches and genetic algorithms. These methods cannot guarantee the most optimal structure and cannot determine the accuracy of a given prediction toward optimality [9–13].

A different approach to pseudoknot prediction adopts dynamic programming to predict the tractable subclass of pseudoknots based on complex thermodynamic models in* O*(*n* ^4^)–*O*(*n* ^6^) time [14–16], making them impractical even for sequences of a few hundred bases long.

Comparative approaches can also be applied to predict pseudoknots and are more reliable than thermodynamic approaches. For example, comparative analysis has revealed the existence of pseudoknots in several RNAs [17]. However, comparative analysis has typically been conducted in an ad hoc manner from an algorithmic point of view.

RNAs fold during transcription from DNA into RNA. Current RNA structure prediction by calculating the global optimal structure does not reflect the dynamic folding mechanism of RNA [18].

Although DP can accurately predict a minimum energy structure within a given thermodynamic model, the native fold is often in a suboptimal energy state that significantly varies from the predicted one [19]. A case may be made that the natural folding process of RNA and the simulated folding of RNA using an evolutionary algorithm, which includes intermediate folds, have much in common [20, 21].

The current study provides a novel report that RNA folding accords with the golden mean characteristic based on the statistical analysis of real RNA secondary structures. The golden mean is also called the golden section or golden ratio. Adolf Zeising found the golden ratio expressed in the arrangement of branches along the stems of plants and of veins in leaves [22]. He extended his research to the skeletons of animals and the branches of their veins and nerves, to the proportions of chemical compounds and geometry of crystals, and even to the use of proportion in artistic endeavors. In these phenomena, he found the golden ratio operating as a universal law. In 2010, the journal* Science* reported that the golden ratio is present at the atomic scale in the magnetic resonance of spins in cobalt niobate crystals [23]. Several researchers have proposed connections between the golden ratio and human genome DNA [24, 25].

Applying this characteristic, we design a golden mean (GM) algorithm by dynamically folding RNA secondary structures according to the golden section points and by forming pseudoknots subsequently folded between nucleotides that did not pair in previous steps.

We implement the method using thermodynamic data and test the performance on PKNOTS and TT2NE data set. For PKNOTS data set, the sensitivity and PPV of the Lhw-Zhu (LZ) and PKNOTS algorithms are increased by 2% to 3% via the preprocessing of the GM method. For TT2NE data set, the GM method indicates good performance in predicting secondary and pseudoknotted structures. The experimental results reflect the folding rules of RNA from a new angle that is close to natural folding.

## 2. Materials and Methods

### 2.1. Structure Prediction

Let sequence *s* = *s*_1_ *s*_2_ ⋯ *s*_*n*_ be a single-stranded RNA molecule, where each base is *s*_*i*_ ∈ {A, U, C, G}, 1 ≤ *i* ≤ *n*. The subsequence *s*_*i*,*j*_ = *s*_*i*_ *s*_*i*_ + 1 ⋯ *s*_*j*_ is a segment of *s*, 1 ≤ *i* ≤ *j* ≤ *n*.

For first approximation, the secondary structure is modeled as follows. If *s*_*i*_ and *s*_*j*_ are complementary bases (A&U, C&G, U&G), then *s*_*i*_ and *s*_*j*_ may constitute a base pair (*i*, *j*). Each base can occur in one base pair, or the set of base pairs can form a matching. The secondary structures are also noncrossing.

Concretely, a secondary structure *S* on *s* is a set of base pairs *S* = {(*i*, *j*)}, where *i*, *j* ∈ {1, 2,…, *n*}, that satisfies the following conditions.

*No Sharp Turns*. The ends of each pair in *S* are separated by at least four intervening bases; that is, if (*i*, *j*) ∈ *S*, then *i* < *j* − 3.

For any pair (*i*, *j*) in *S*, (*i*, *j*)∈{(A, U), (C, G), (U, G), (U, A), (G, C), (G, U)}.

*S* is a matching: no base appears in more than one pair.

*Noncrossing Condition*. If (*i*, *j*) and (*k*, *l*) are two pairs in  *S*, then they are compatible; that is, they are juxtaposed (e.g., *i* < *j* < *k* < *l*) or nested (e.g., *i* < *k* < *l* < *j*).

Base pair and internal unpaired bases construct loops. If (*i*, *j*) and (*i* + 1, *j* − 1) ∈ *S*, base pairs (*i*, *j*) and (*i* + 1, *j* − 1) constitute stack (*i*, *i* + 1 : *j* − 1, *j*) and *m*  (≥1) consecutive stacks form the helix (*i*, *i* + *m* : *j* − *m*, *j*) with the length of *m* + 1.

If base pairs (*i*, *j*) and (*k*, *l*) are parallel (*i* < *j* < *k* < *l*  or  *k* < *l* < *i* < *j*) or nested (*i* < *k* < *l* < *j*  or  *k* < *i* < *j* < *l*), then base pairs (*i*, *j*) and (*k*, *l*) are compatible; otherwise, they are incompatible. Such an incompatible structure is knownas a pseudoknot (e.g., *i* < *k* < *j* < *l*). More complex pseudoknots may occur if three or more base pairs cross each other.

The concept of the domain was first proposed in 1973 by Wetlaufer after X-ray crystallographic studies on hen lysozyme [26] and papain [27] and after limited proteolysis studies on immunoglobulins [28, 29]. Wetlaufer defined domains as stable units of protein structure that can fold autonomously. Domains have been previously described as units of compact structure [30], function and evolution [31], and folding [32].

Each domain forms a compact 3D structure and is often independently stable and folded. Most domains have less than 200 residues with an average of approximately 100 residues [33, 34].

A domain *D*(*i*′, *j*′) consists of all (*i*′, *j*′) that satisfy (*i*′, *j*′) ∈ *D*(*i*, *j*); then *i* < *i*′ < *j*′ < *j*. A base pair can only occur in one domain.

A domain is closed by a helix or pseudoknot (Figure 3). A subdomain is an independently stable part of a domain. If the closed helix or pseudoknot of a domain is deleted, its subdomain will become a domain.

By convention, single strands of DNA and RNA sequences are written in 5′-to-3′ direction. RNAs fold during transcription from DNA into RNA. The subsequence *s*_*i*,*j*_ begins to transcribe from the 5′-end, that is, *s*_*i*_, and terminates transcription at the 3′-end, that is, *s*_*j*_ (Figure 3). The helix (*i*, *i* + *m* : *j* − *m*, *j*) is completely folded after the transcription of *s*_*j*_. We can determine that the 3′-end of the helix (*i*, *i* + *m* : *j* − *m*, *j*) and the domain *D*(*i*, *j*) is the 3′-end of subsequence *s*_*i*,*j*_. Let *L* be the sequence length. The length ratio of the 3′-end of the helix (*i*, *i* + *m* : *j* − *m*, *j*) and the domain *D*(*i*, *j*) to the sequence *s*_1,*L*_ is the ratio of *j* to *L*. This study determines the characteristic of the 3′-end and the length of domain in the sequences.

### 2.2. Characteristic of Golden Section

We compare the structures of the test set of all 480 sequences (nonfragment and nonredundant) from RNA STRAND with secondary and pseudoknotted structures, which are validated by NMR or X-ray.

**Figure 1:**
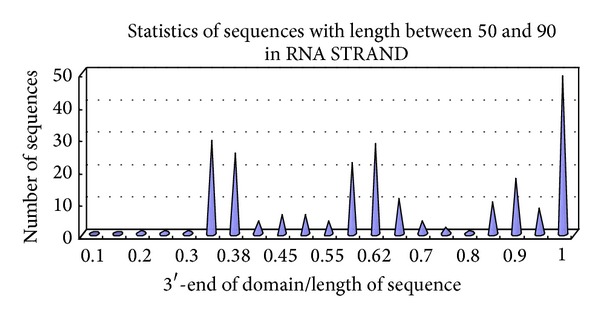
Distribution of domains for sequences with lengths between 50 and 90. (a) For all NMR- or X-ray-validated data on 55 nonfragment and nonredundant sequences with lengths between 50 and 90 from RNA STRAND, except for synthetic RNA, the length ratio of the 3′-end of the domains to the sequence is computed and summarized. If one sequence has only one domain, subdomains are selected. (b) The *x*-axis represents the length ratio of the 3′-end of the domain to the sequence, and the *y*-axis represents the number of sequences.

**Figure 2:**
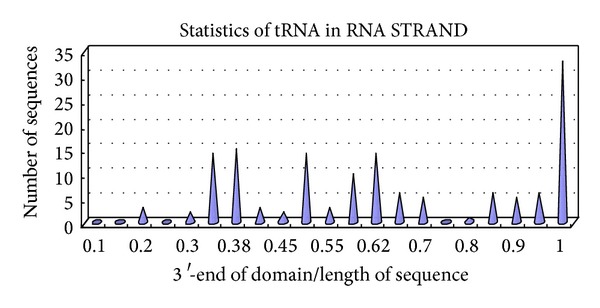
Distribution of domains for tRNA. (a) For all NMR- or X-ray-validated data on 35 nonfragment and nonredundant sequences with lengths more than 50 from RNA STRAND, the length ratio of the 3′-end of the domains to the sequence is computed and summarized. If one sequence has only one domain, subdomains are selected. (b) The *x*-axis represents the length ratio of the 3′-end of the domain to the sequence, and the *y*-axis represents the number of sequences.

The results of statistical analysis on these real secondary structures are shown in Figures 1 and 2 and Tables 1, 2, 3, and 4. In Tables 1 and 2, Num represents the number of domains, group is the 3′-end of group, Ratio 1 is the ratio of Group 1 to Group 2, and Ratio 2 is the ratio of Group 2 to Group 3. In Figures 1 and 2, the *x*-axis represents the length ratio of the 3′-end of domain to the sequence, and the *y*-axis represents the number of sequences. The number of complementary bases to form a helix at point *x* in the final structure is insufficient. Thus, we enlarge point *x* to region *x*, and the corresponding point *y* in the *y*-axis with *x* in the *x*-axis represents the number of sequences in the region of [*x* − 0.4, *x*].

For 16S rRNA, 26 sequences belong to RNA STRAND. The statistical result is shown in Table 1. The number of domains varies from 4 to 8. The length ratio of the first domain to the sequence is shown as Ratio 2. The length ratio of the first subdomain to the first domain is shown as Ratio 1. The two ratios are both close to 0.618.

For example, four domains belong to sequence PDB_00409, namely,* D* (3, 726),* D* (731, 1063),* D* (1084, 1129), and* D* (1150, 1171). In addition, the subdomains of* D* (23, 437) are* D* (443, 705) and* D* (1150, 1171). We can divide the sequence into three groups, namely, 1–442, 1–730, and 731–1174, which represent the length of the entire sequence. The ratio of Group 1 to Group 2 is 0.61, and the ratio of Group 2 to Group 3 is 0.62 to 0.38.

For long 5S rRNA, 23S rRNA, GI Intron, rRNA, and tRNA in RNA STRAND, the statistical results are shown in Table 2. The number of domains varies from 1 to 6. The length ratio of the first subdomain to the first domain for 5S rRNA is 0.58 and that of GI Intron is close to 0.37. The length ratio of the second domain to the sequence for tRNA and other rRNA is close to 0.38. The length ratio of the third domain to the sequence for tRNA is close to 0.6 and that of the fourth domain to the sequence for other rRNA is 0.59. For 23S rRNA, the subdomains are* D* (16, 2625),* D* (2630, 2788), and* D* (2647, 2726). The subdomains of* D* (16, 2625) are* D* (29, 476),* D* (579, 1261),* D* (1269, 2011), and* D* (2023, 2040).* D* (16, 2625) is a pseudoknot, Ratio 1 is the ratio of 1261 to 2040, and Ratio 2 is 2040 to the sequence length.

For short RNAs, sequences with lengths less than 50 have generally only one domain and one or two simple helices. For sequences with lengths between 50 and 90, except for synthetic RNA, the folding 3′-end of domains and subdomains is centered on three regions (Figure 1). The sequences have one to three domains and that with only one domain has two or three subdomains. The sequences fold on three regions. The first folds in the region of 0.35*L* to 0.38*L*, the second in the region of 0.6*L* to 0.618*L*, and the third in the region of 0.9*L* to 1.0*L*. Among 33 sequences, 25, 28, and 49 have helix 3′-ends located in points 0.382*L*, 0.618*L*, and* L*, respectively.

For tRNAs with lengths more than 50, the folding 3′-end of domains and subdomains is centered on four regions (Figure 2). The sequences have one to three domains and that with only one domain has two or three subdomains. Among 35 sequences, 15, 14, 14, and 33 have domain or subdomain 3′-ends located at points 0.382*L*, 0.5*L*, 0.618*L*, and* L*, respectively.

The data corresponding to Figure 1 are shown in Table 3.

The data corresponding to Figure 2 are shown in Table 4.

The sequences tend to fold twice or thrice. For the sequences that fold twice, the first folds in the region of 0.5*L* and the second in the region of* L*. For the sequences that fold thrice, the first folds in the region of 0.35*L* to 0.38*L*, the second in the region of 0.6*L *to 0.618*L*, and the third in the region of* L*.

In mathematics and the arts, two quantities belong to the golden ratio if their ratio is the same as the ratio of their sum to their maximum. Expressed algebraically, for quantities *a* and *b* with *a* > *b*, *a*/*b* = (*a* + *b*)/*a* = phi = 1.618, and the quantities are 0.382 (*a* + *b*) and 0.618 (*a* + *b*). These quantities increase several unique ratios, including 0.618, 0.382, and 1.618, that is, the golden ratio. These ratios exist throughout nature, from population growth to the physical structure within the human brain, the DNA helix, many plants, and even the cosmos itself. The golden ratio is also called the golden section or golden mean.

The golden mean and the numbers of the Fibonacci series (0, 1, 1, 2, 3, 5, 8,…) have been used with significant success in analyzing and predicting stock market motion. Elliott presented wave theory, in which the frequent wave relationships are golden ratios (19.1%, 23.6%, 38.2%, 50%, 61.8%, and 80.9%). It has a striking similarity to RNA folding. We can determine that 0.382, 0.5, and 0.618 are the only important RNA folding points (Tables 1 to 4). The above results of statistical analysis also confirm this view. Almost all sequences are folded at* L*, and approximately half of the sequences are folded at points 0.382*L*, 0.5*L*, and 0.618*L*. For long sequences, the other two key fold points 0.236*L* and 0.809*L* may be found, which are 0.382*L* × 0.618 and 0.5*L* × 1.618.

Therefore, these points would be closer to natural folding and obtain higher accuracy than before if RNA is dynamically folded according to the above golden points.

### 2.3. Dynamic Algorithm

RNAs fold through a hierarchical pathway, in which the helices and loops are rapidly formed as secondary structures and the subsequent slow folding of the 3D tertiary structures would consolidate the secondary structures [20, 21]. RNAs also fold during transcription from DNA into RNA. Therefore, we first compute the secondary structure and then predict pseudoknotted structures. We fold RNA secondary structures as DNA is transcribed into RNA. The length of RNA sequences is gradually increased according to the above golden points, and only reliable helices are accepted.

**Figure 3:**
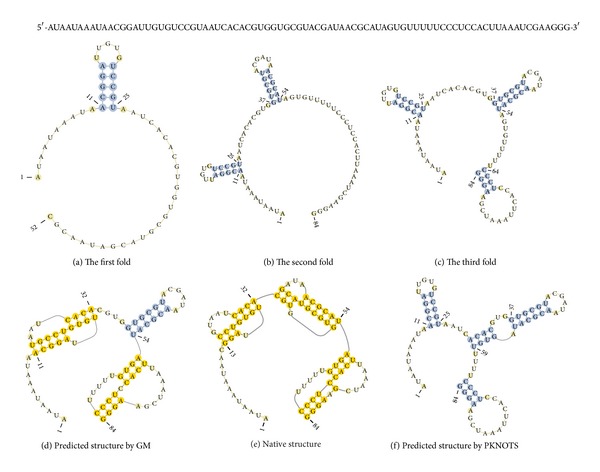
Computation of sequence TMVup. (a) Status after the first fold of the GM algorithm. (b) Status after the second fold of the GM algorithm. (c) Intermediate result of the last step of the GM algorithm. (d) Final fold and predicted structure by GM. (e) Native structure of TMVup. (f) Final fold and predicted structure by PKNOTS. (g) The top shows the sequence of TMVup.

For example, we first fold the subsequence *s*_1,52_ of TMVup with a length of 52, that is, 0.618*L*, and form the helix (11, 15: 21, 25) (Figure 3(a)). Then, we fold the subsequence *s*_1,84_ of TMVup with a length of 84, that is, 0.618*L *× 1.618 =* L*, and form the helix (37, 42: 49, 54) (Figure 3(b)). Finally, we fold two pseudoknots (Figure 3(d)). Figure 3(c) shows the intermediate result of the last step, where the helix is formed (64, 67: 81, 84).

One pseudoknot can consist of one helix and two subsequences, and one of the two is included in the helix. Therefore, we can compute the crossing of two subsequences. Pseudoknots consist of one internal and one external subsequence of helix, and secondary structures consist of two external subsequences of helices. The residual sequence consists of six sequences after secondary structure folding (Figure 3(c)). The subsequences *s*_16,20_ and *s*_26,36_ form the helix (17, 20: 29, 32) (Figure 3(d)), and the subsequences *s*_68,80_ and *s*_55,63_ form the helix (55, 58: 69, 74) (Figure 3(d)). The helices (55, 58: 69, 74) and (64, 67: 81, 84) form one pseudoknot, and the helices (17, 20: 29, 32) and (11, 15: 21, 25) form another pseudoknot.

The sketch of the GM algorithm is as in Algorithm 1.

The method uses a dynamic programming strategy to compute the secondary structure values of *Z*(*i*, *j*) for all *i* and *j* in the start subsequence. The subsequence is then iteratively lengthened to the next golden point to compute the secondary structure values of *Z*(*i*, *j*) for all *i* and *j* in the new subsequence. Finally, the pseudoknots are computed by the crossing of two subsequences, and the values of *Z*(*i*, *j*) are provided for all *i* and *j* in the entire sequence. In this study, we use our previous LZ algorithm to compute pseudoknotted structures [16].

For the sequence of TMVup, the startLen is 0.618*L*, that is, 52. We fold the subsequence *s*_1,52_ and select the helix* H* (11, 15: 21, 25) with the maximum value; assign *i* as another golden point 1.618 × 52, that is,* L*, and select the helix* H* (37, 42: 49, 54). The LZ algorithm computes the residual subsequence and forms the last structure. The ratio of the latter golden point to the former one is 1.618, and the length of the start subsequence should be between 40 and 70.

The time complexity of steps 1.1 to 1.5 is* O*(*n* ^3^), and the number of iterative computations is less than 10 for sequences with lengths less than 1000. Therefore, the first step takes* O*(*n* ^3^) time. In this study, the time complexity of step 2 is less than* O*(*n* ^5^), which is similar to that of the LZ algorithm. Therefore, the time complexity of the GM algorithm is maintained as* O*(*n* ^5^).

## 3. Results and Discussion

To illustrate the effect of our algorithm, tests are divided into two parts: one for pseudoknotted sequences and another for mixed data of pseudoknot-free and pseudoknotted sequences. We select two data sets. One is TT2NE data set to test pseudoknotted structures. This data set contains 47 pseudoknotted sequences from PseudoBase and PDB. Another is PKNOTS data set to test secondary and pseudoknotted structures. This data set includes 116 sequences, including 25 tRNA sequences randomly selected from the Sprinzl tRNA database, HIV-1-RT-ligand RNA pseudoknots, and some viral RNAs.

The accuracy of an algorithm is measured by both sensitivity and PPV. Let RP (real pair) be the number of base pairs in the real RNA structure, TP (true positive) the number of correctly predicted base pairs, and FP (false positive) the number of wrongly predicted base pairs. Burset and Guigo [35] defined SE (sensitivity) as TP/RP and PPV (positive predictive value) as TP/(TP + FP) in 1996.

### 3.1. Results of PKONTS Data Set

In this section, we test the PKNOTS data set, which has become the benchmark dataset to predict RNA structures and present the prediction results of our method compared with the PKNOTS algorithm [14] and our previous LZ algorithm [16].

To explore the effect of the GM method in different models, we test two models and compare the difference before and after GM processing. First, we run PKONTS and LZ algorithm on the PKNOTS data set and obtain the output of the results. We then run the first step of the GM method to form the frame of secondary structures by dynamically folding sequences at the golden points and select one stable helix with the minimum energy at each fold. Subsequently, we obtain the partially folded sequences as the input of PKONTS and LZ algorithm and run them with the same energy model and parameters as above. We fold all sequences of the test set and obtain the results. The test results are shown in Table 5.

For each algorithm, the percentages of sensitivity and PPV are shown in Table 5. The value is the usual average of sensitivity and PPV values of all sequences. The detailed results are displayed as Supplementary data in Supplementary Material available online at http://dx.doi.org/10.1155/2014/690340.

The improved PKNOTS algorithm is compared with the PKNOTS algorithm in both sensitivity and PPV (Table 5). The improved PKNOTS algorithm increases the sensitivity from 82.8% to 85.5% and improves the PPV from 78.9% to 80.8%.

The improved LZ algorithm is compared with the LZ algorithm in both sensitivity and PPV (Table 5). The improved LZ algorithm increases the sensitivity from 84.8% to 87.7% and improves the PPV from 80.7% to 82.8%.

The improved LZ and PKNOTS algorithms both have 2% to 3% higher sensitivity than the LZ and PKNOTS algorithms, respectively. The improved LZ algorithm outperforms the PKNOTS algorithm by 4.9%. The tests of improved PKNOTS and improved LZ indicate that the GM method may also be applied to other algorithms of RNA structure prediction to improve the prediction sensitivity and reduce the predicted redundant base pairs.

Both of the improved LZ and PKNOTS algorithms increase the accuracy of many sequences (e.g., Bioton, DF0660, DG7740, DI1140, DP1780, DV3200, and DY4840), from which we can determine the influence of the golden mean characteristic.

**Figure 4:**
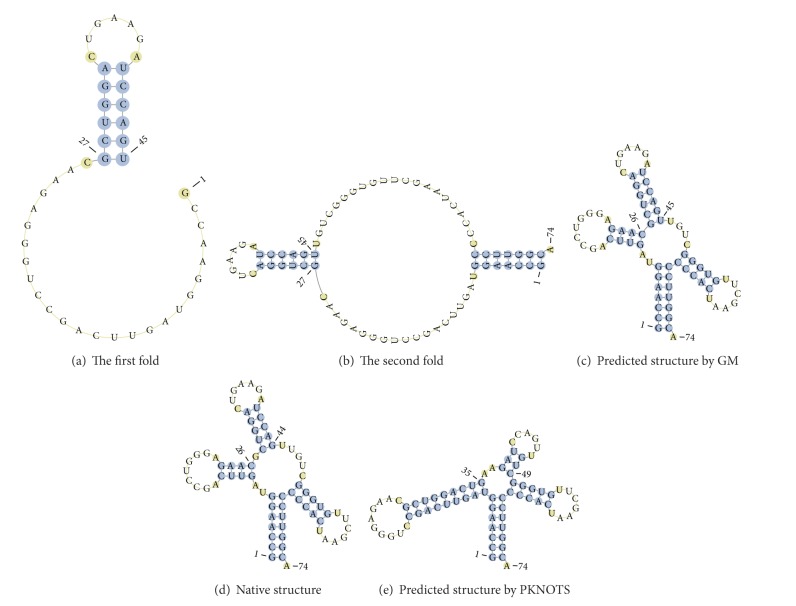
Predicted structure of sequence DF0660. (a) Status after the first fold of GM algorithm. (b) Status after the second fold of GM algorithm. (c) Final fold and predicted structure by GM. (d) Native structure of DF0660. (e) Final fold and predicted structure by PKNOTS.

For example, in GM, the first DF0660 sequence is folded at golden point 0.618*L* and forms the helix (27, 32: 40, 45), which exists in real structure except for one base pair (27, 45) (Figure 4(a)). This sequence is then folded at point* L* and selects the helix (2, 7: 67, 72), which exists in real structure (Figure 4(b)). Subsequently, this sequence selects two helices [(10, 13: 23, 26) and (50, 54: 63, 67)], which exist in real structure (Figure 4(c)).

However, the PKNOTS algorithm selects two helices [(8, 16: 27, 35) and (38, 40: 47, 49)], which do not exist in real structure (Figure 4(e)). Only the form of helix (27, 32: 40, 45) at the first golden point prevents the formation of a large helix (8, 16: 27, 35) in GM.

For pseudoknotted structure in GM, sequence TMVup is folded to form two pseudoknots and one helix (37, 42: 49, 54), which exist in real structure, except for one redundant base pair (11, 25) (Figure 3(d)). The sequence also missed one helix (34, 36: 43, 45), which is one pseudoknot (Figure 3(d)).

In the PKNOTS algorithm, sequence TMVup is folded to form four helices, three of which are equal to those in GM, but one helix (29, 33: 56, 59) is redundant (Figure 3(f)). This sequence also missed all three pseudoknots. Only the form of the helix (29, 33: 56, 59) prevents the form of pseudoknots.

Further statistical analysis shows that the selected helices by the first step of GM basically belong to the real structures and control the folding pathway. This finding is precisely ascribed to the GM processing before PKONTS and LZ improve the accuracy.

### 3.2. Results of TT2NE Data Set

In this section, we test the TT2NE data set, which includes 47 sequences from PseudoBase and PDB, which is also a subset of those used in the HotKnots.

We present the prediction results of our method compared with those of HotKnots [9], McQfold [10], ProbKnot [11], TT2NE [13], Mfold [36], and LZ [16]. MFold is restricted to secondary structures that are free of pseudoknots, whereas others can result in any topology of pseudoknot.

This set includes most of the sequences where HotKnots has been tested and shown to perform better than ILM and PKnots-rg. Thus, we will not compare GM to these latter algorithms.

The total number of base pairs to be predicted in this set is 1115. Mfold, HotKnots, McQfold, ProbKnots, TT2NE, LZ, and GM predicted 618, 671, 740, 669, 870, 785, and 798, respectively. The total numbers of predicted base pairs are 1024, 1019, 991, 1041, 1146, 1102, and 1112, respectively.

The sensitivity of GM is better than that of the other algorithms, except for TT2NE, either on the average 1 or on the average 2 (Table 6). The PPV of GM is better than that of the other algorithms, except for TT2NE and McQfold, either on the average 1 or on the average 2.

However, TT2NE has shown the most feasible value after several times folding with different parameters. Thus, TT2NE is not the autocomputing value. When given new sequences, we do not know which result should be selected.

The part of computing pseudoknots in GM is the same as that in the LZ algorithm, and the difference between them lies in the preprocessing of secondary structures. The performance of GM is better than that of the LZ algorithm, either on the average 1 or on the average 2. GM improves the sensitivity of sequences Bs_glmS, EC_S15, and HDV_antigenomic from 36, 59, and 44 to 51, 100, and 84, respectively. GM also improves the PPV of these sequences from 57, 63, and 34 to 67, 74, and 64, respectively. However, for sequences AMV3 and BVDV, the performance of GM is only below LZ.

The number of predicted genera by GM is better than that by other algorithms, except for TT2NE (Table 6). All Mfolds predictions have genus 0 because Mfold generates only structures without pseudoknots.

However, TT2NE predicted more redundancy genera than other algorithms. For example, GLV_IRES, R2_retro_PK, and 1y0q sequences have only one native pseudoknot, but two pseudoknots are predicted by TT2NE. Bs_glmS has two native pseudoknots, but three are predicted by TT2NE.

The percentages of sensitivity and PPV of each algorithm are shown. The column Gen indicates the predicted number of genera, and GR is the redundancy number in the predicted genus, which is more than the number of native genera. Average 1 is the total number of base pairs that are correctly predicted in the entire database divided by the corresponding total number of native base pairs (average sensitivity) and the total number of predicted base pairs (average PPV). Average 2 is the usual average of sensitivity and PPV values of all sequences. The detailed results are displayed as Supplementary data.

## 4. Conclusions 

In this study, we provide a novel report that RNA folding accords with the golden mean characteristic based on the statistical analysis of real RNA secondary structures. The folding 3′-end points of the sequence are almost close to 0.382*L*, 0.5*L*, 0.618*L*, and* L*. These points are the important golden sections of sequence. Applying this characteristic, we design a GM algorithm by dynamically folding RNA secondary structures according to the above golden section points and by forming pseudoknots with the crossing of subsequences. We implement the method using thermodynamic data and test its performance on PKNOTS and TT2NE data sets.

For PKNOTS data set, we preprocess the sequence with the first step of GM and then obtain the output of the partially folded sequence as the input of the PKNOTS and LZ algorithms. Subsequently, the two algorithms improve by 2% to 3%. The reason is that the partial folded sequence forms its structural frame. In other words, the folding at the golden points controls the folding pathway and subsequently prevents the formation of some redundant structures.

Preprocessing of GM may also be applied to other algorithms of RNA structure prediction to improve the prediction accuracy and to reduce the predicted redundant base pairs.

For TT2NE data set, the sensitivity and number of predicted pseudoknots of GM are better than those of Mfold, HotKnots, McQfold, ProbKnot, and LZ. The PPV of GM is better than that of Mfold, HotKnot, ProbKnot, and LZ. These findings indicate that the GM method has good performance in predicting secondary and pseudoknotted structures. The sensitivity and PPV of the GM algorithm surpass those of most algorithms of RNA structure prediction. The experimental results reflect the folding rules of RNA from a new angle that is close to natural folding.

The performance of long sequence needs to be further explored, and parameters for improvement and testing in large data sets to support web service are topics for future studies.

## Supplementary Material

PKNOTS data set includes 116 sequences, that is 25 tRNA sequences randomly selected from Sprinzl tRNA database, HIV-1-RT-ligand RNA pseukonts, and some viral RNAs. Column len and base pair indicate the length and the number of the native base pairs of sequence. For each algorithm, the sensitivity and specificity are shown. Average is is the usual average of sensitivity and specificity values reported in the table.
TT2NE data set has 47 sequences from PseudoBase and PDB. In column ex, X indicates whether the native has been found experimentally. For each algorithm, the sensitivity and specificity are shown. Column g indicates the genus of the native structure and gT, gHK, gMQ, gPK, gLZ and gGM, are the respective genii of the predictions of TT2NE, HotKnots, McQfold, ProbKnot, LZ and GM. All Mfolds prediction have genus 0, as Mfold generates only structures without pseudoknots. Average 1 is the total number of base pairs correctly predicted in the whole database divided by respectively the total number of native base pairs (average sensitivity) and the total number of base pairs predicted (average specificity). Average 2 is the usual average of sensitivity and specificity values reported in the table (values used to perform the t-test).
Notice: TT2NE is shown the best value after several times folding with different parameters, so it is not the auto-computing value.

## Figures and Tables

**Algorithm 1:**
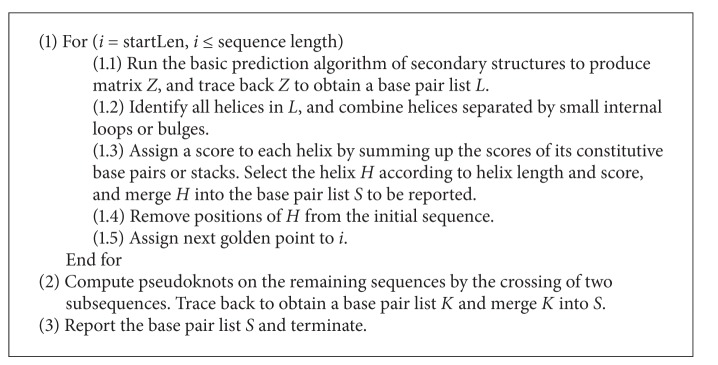


**Table 1:** Distribution of domains for 16S rRNA.

Sequence ID	Length	Num	Group 1	Group 2	Group 3	Ratio 1	Ratio 2
PDB_00409	1174	4	442	730	1174	0.61	0.62
PDB_00456	1514	4	548	896	1514	0.61	0.59
PDB_00478	1513	4	545	893	1513	0.61	0.59
PDB_00643	1688	9	559	913	1688	0.61	0.54
PDB_00645	1533	4	559	915	1533	0.61	0.60
PDB_00703	1466	4	520	868	1466	0.60	0.59
PDB_00769	1526	4	559	915	1533	0.61	0.60
PDB_00791	1530	4	562	916	1533	0.61	0.60
PDB_00811	1521	5	545	893	1521	0.61	0.59
PDB_00812	1522	5	545	893	1522	0.61	0.59
PDB_00813	1522	5	545	893	1522	0.61	0.59
PDB_00814	1522	5	545	893	1522	0.61	0.59
PDB_00946	1673	6	545	895	1673	0.61	0.53
PDB_00952	1673	8	545	895	1673	0.61	0.53
PDB_01015	1527	5	546	894	1527	0.61	0.59
PDB_01039	1511	4	545	893	1511	0.61	0.59
PDB_01088	1716	7	532	880	1716	0.60	0.51
PDB_01101	1667	5	545	893	1667	0.61	0.54
PDB_01103	1793	6	545	893	1667	0.61	0.54
PDB_01105	1694	5	545	893	1667	0.61	0.54
PDB_01107	1505	4	545	893	1533	0.61	0.58
PDB_01139	1687	6	545	893	1673	0.61	0.53
PDB_01198	1660	5	545	893	1667	0.61	0.54
PDB_01240	1685	5	545	893	1667	0.61	0.54
PDB_01268	1528	5	545	893	1522	0.61	0.59
PDB_01269	1529	5	545	893	1522	0.61	0.59

**Table 2:** Distribution of domains for other long RNAs.

Sequence ID	Type	Length	Num	Group 1	Group 2	Group 3	Ratio 1	Ratio 2
PDB_00030	5S rRNA	120	1	69	120		0.58	
PDB_00082	GI Intron	315	1	116	315		0.37	
PDB_00140	GI Intron	314	1	116	316		0.37	
PDB_00398	tRNA	380	5	148	228	408	0.38	0.6
PDB_01144	Other rRNA	408	6	151	240	408	0.37	0.59
PDB_00029	23S RRNA	2904	1	1261	2040	2904	0.62	0.70

**Table 3:** Distribution of domain and subdomain for sequences with lengths between 50 and 90.

Ratio	0.05	0.1	0.15	0.2	0.25	0.3	0.35	0.382	0.40	0.45	0.5	0.55	0.6	0.618	0.65	0.7	0.75	0.8	0.85	0.9	0.95	1

Number	1	0	0	1	1	1	29	25	4	6	6	4	22	28	11	4	2	1	10	17	8	49

**Table 4:** Distribution of domains for tRNA.

Ratio	0.05	0.1	0.15	0.2	0.25	0.3	0.35	0.382	0.40	0.45	0.5	0.55	0.6	0.618	0.65	0.7	0.75	0.8	0.85	0.9	0.95	1

Number	1	0	0	3	0	2	14	15	3	2	14	3	10	14	6	5	0	1	6	5	6	33

**Table 5:** Prediction results of improved PKNOTS and LZ algorithm.

RNAs	SE	PPV
PKNOTS-1.05	82.8	78.9
Improved PKNOTS-1.05	85.5	80.8
LZ	84.8	80.7
Improved LZ	87.7	82.8

**Table 6:** Prediction results of TT2NE data set.

	Mfold		HotKnots		McQfold		ProbKnot		TT2NE		LZ		GM	
	SE	PPV	SE	PPV	SE	PPV	SE	PPV	SE	PPV	SE	PPV	SE	PPV
Average 1	55	60	60	67	66	75	60	64	78	76	70	71	72	72
Average 2	50	58	63	67	68	77	54	63	81	80	73	73	75	74
Sum	0	0	18	0	25	0	3	0	47	4	28	0	31	0
	**Gen**	**GR**	**Gen**	**GR**	**Gen**	**GR**	**Gen**	**GR**	**Gen**	**GR**	**Gen**	**GR**	**Gen**	**GR**
